# Epidemiology and fungal species distribution of onychomycosis and other superficial mycoses in the coastal city of Ningbo, China

**DOI:** 10.3389/ffunb.2026.1716554

**Published:** 2026-04-02

**Authors:** Yao Lu, Pingping Qiu, Yijun Mo, Minhong Zhu, Xumei Wang, Xingbei Weng

**Affiliations:** 1 Department of Laboratory Medicine, The First Affiliated Hospital of Ningbo University, Ningbo First Hospital, Ningbo, Zhejiang, China; 2 Department of Interventional Center, The First Affiliated Hospital of Ningbo University, Ningbo First Hospital, Ningbo, Zhejiang, China; 3 Department of Laboratory Medicine, Ninghai County Third Hospital, Ningbo, Zhejiang, China

**Keywords:** dermatophytes, onychomycosis, SFIs, superficial fungal infections, superficial mycoses, tinea

## Abstract

**Background:**

Superficial fungal infections (SFIs) are among the most common skin diseases worldwide; however, the pathogenic species of SFIs vary among geographical areas. The purpose of this study was to evaluate the epidemiologic profile of superficial fungal infections with a focus on onychomycosis in the Department of Laboratory Medicine of Ningbo First Hospital.

**Methods:**

A total of 1309 patients with superficial mycoses who visited our hospital between March 2023 and October 2024 were selected. Scraping of the lesion was performed via a sterilization scraper. A portion of each sample was used for direct examination, and the other portion was used for cultivation. For the culture results, microscopic morphology and automatic rapid microbial matrix-assisted laser desorption/ionization time-of-flight mass spectrometry (MALDI-TOF MS) were used as a routine examination. For fungi that routine examinations couldn’t identify, further sequencing of the rDNA-ITS region was carried out for atypical strains to the species level.

**Results:**

Patients were between 1 year and 93 years old, and the peak incidence was in the 21–30 years age group. More superficial mycoses were found in women (63.94%) than in men (36.06%). The causative agents identified were: dermatophytes (32.85%), yeasts (49.20%) and non-dermatophyte molds (NDM) (17.95%). Positive cultures of dermatophytes were obtained from 430 patients: *Trichophyton* spp. (89.07%) was predominant, followed by *Microsporum* spp. (10.93%). Yeasts were isolated from 644 patients, and *Candida* spp. (93.01%) were the major pathogens, followed by *Trichosporon* spp. (6.99%). Among the 1309 patients, nails were the largest source of SFIs (81.67%), followed by glabrous skin (6.04%) and feet (4.20%). *Candida* spp. were the most prevalent species in cases of onychomycosis (47.05%), followed by *Trichophyton* spp. (29.09%) and *Aspergillus* spp. (16.65%).

**Conclusion:**

Our study revealed that nails constituted the largest source of SFIs in Ningbo, and women were more likely to have onychomycosis than men were. Moreover, we found that *Candida* spp. was the most prevalent species in cases of onychomycosis, and *Trichophyton rubrum* was responsible for the majority of dermatophyte-associated infections.

## 1 Introduction

Superficial fungal infections (SFIs) are the most common fungal disease and the most common infectious disease in dermatology. They are limited to the outermost layers of the skin and appendages (Chen et al., 2018). SFIs are caused mostly by dermatophytes, yeasts, and to a lesser extent, non-dermatophyte filamentous fungi (NDFF) such as *Fusarium* and *Aspergillus (*Khodadadi et al., 2021). Globally, approximately 20–25% of the population is affected by superficial mycosis. And dermatophyte infections, which is also referred to as ringworms or tinea, is the most prevalent disease of SFIs (Havlickova et al., 2008; Jaishi et al., 2022). Dermatophytes are a group of filamentous fungi that infect keratin-rich tissues and can be categorized into anthropophilic, zoophilic, and geophilic dermatophytes on the basis of host preferences and ecological niches (Ameen, 2010; Deng et al., 2023). They can adhere to the surface of keratinized tissues and release proteolytic enzymes during invasion, which not only allows their survival but also ascertain the persistence of infection in the host (Gupta et al., 2023; Dubljanin et al., 2024). Dermatophytes have recently been classified into seven genera, namely, *Trichophyton*, *Microsporum*, *Arthroderma*, *Epidermophyton*, *Lophophyton*, *Paraphyton* and *Nannizzia (*Mehrmal et al., 2020). Dermatophytosis is a disease caused by dermatophytes in both animals and humans and is generally chronic in nature. According to the Latin term that designates the anatomic site of infection, the most common clinical manifestations are tinea capitis (scalp), tinea corporis (body), tinea manuum (hand), tinea cruris (groin), tinea pedis (foot) and tinea unguium (nail) (Kovitwanichkanont and Chong, 2019). Proper identification of etiological agents and their prevalence are critical for the treatment and control of dermatophytosis (Begum et al., 2020). In addition to dermatophytes, yeasts of the genus *Candida*(*C*.) often cause SFIs, especially *C. albicans*. *Candida* yeasts are the host commensal flora; therefore, local skin conditions, medical or disease-related immunosuppression, endocrine disorders or compromised blood flow can lead to SFIs (Taudorf et al., 2019; Howell, 2023).

With the wide application of broad−spectrum antibiotics, glucocorticoids, immunosuppressants and anti−tumor drugs, the prevalence rate of SFIs is increasing annually (Wang et al., 2020). Ningbo is an important port city on the southeast coast of China with high population density and unique geographical conditions. In Ningbo, our hospital is the only one that performs superficial fungal cultures, so few studies on SFIs have been conducted in this city.

## 2 Materials and methods

### 2.1 Clinical information

A total of 1309 patients with superficial mycoses who visited our hospital between March 2023 and October 2024 were selected. Among them, 472 patients were males and 837 patients were females. The male to female ratio was 4:7, with the youngest being 1 year old and the oldest being 93 years old. Among their samples, 1069 from nails (81.67%), 220 from skin (16.80%), and 20 from hair (1.53%) lesions were collected. This study was approved by the Ethics Committee of Ningbo First Hospital(Approval No. 2024RS238-01).

### 2.2 Fungal culture and identification

Scraping of the lesion was performed via a sterilization scraper. A portion of each sample was used for direct examination, and the other portion was used for cultivation. Direct examination was performed for all the samples. Clinical samples were stained with calcofluor white staining to help identify suspicious fungal lesions via fluorescence microscopy. We defined the presence of hyphae on fluorescence microscopy as the criterion to differential invasive infection from colonization, as *Candida* yeasts can colonize certain anatomical sites (e.g., nails) without causing disease. For fungal cultivation, samples were inoculated on the surface of Sabouraud Dextrose Agar (SDA) supplemented with chloramphenicol and cycloheximide, and incubated at 28 °C for 2 weeks and examined three times a week. Preliminary identification was carried out according to the characteristics of the colony morphology and pigment produced, growth speed, and morphology of the spores and hyphae under a microscope. For non-dermatophyte molds (e.g., *Aspergillus*), we distinguished true infection from environmental contamination through isolating the same fungal species from repeated samples collected from the same patient (Schelenz et al., 2025). To further determine the culture results, automatic rapid microbial matrix-assisted laser desorption/ionization time-of-flight mass spectrometry (MALDI-TOF MS) (bioMerieux VITEK MS) was used to identify fungal species as a routine supplementary examination. For those fungi that cannot be identified by the conventional detection methods described above, fungal total DNA was extracted, and further sequencing of the rDNA-ITS region was carried out for atypical strains to the species level.

### 2.3 Statistical methods

The database was set up for data processing and data analysis via Excel tables, and the chi-square test was performed via SPSS 23.0 software. The difference was statistically significant at *p* < 0.05.

## 3 Results

Among the 1309 patients with superficial mycoses, 472 were males and 837 were females, and the patients were between 1 year and 93 years old. Females had a higher prevalence of SFIs than males (63.94% *vs*. 36.06%). The proportion of patients in each age group was highest in the 21–30 and 31–40 age groups, with 272 (20.78%) and 257 (19.63%) patients, respectively, accounting for 40.41% of the total patients. The analysis of patients with SFIs at different ages revealed that in the 0–10 years age group (χ2 = 14.627, p<0.001), males had a greater prevalence than females did, and in the 11–20 years age group (χ2 = 6.319, p=0.012), 51–60 years age group (χ2 = 13.019, p<0.001) and ≥61 years age group (χ2 = 5.384, p=0.020), females had a greater prevalence than males did. The difference was statistically significant, as shown in **Table 1**.

**Table 1:** Distribution of SFIs according to gender and age.

Age in years	Male	Female	χ2	p
0-10	36	25	14.627	0.000
11-20	46	50	6.319	0.012
21-30	101	171	0.172	0.678
31-40	85	172	1.235	0.266
41-50	65	144	2.651	0.103
51-60	61	175	13.019	0.000
≥61	78	100	5.384	0.020
Total	472	837		

The main body site affected by superficial mycosis was the nails (81.67%), followed by the glabrous skin (6.04%) and feet (4.20%). The proportion of females with onychomycosis was significantly greater than that of males (χ2 = 16.687, p<0.001), the proportion of males with inguinocrural fungal infections was significantly greater than that of females (χ2 = 12.466, p<0.001), and there was no significant difference in the proportion of males and females with other body site SFIs, as shown in **Table 2**.

**Table 2:** Comparison of main clinical types of SFIs in patients of different sex.

Gender	Number of cases	Nails	Feet	Glabrous skin	Groin	Scalp	Hands	Face
Male	472	358	26	34	24	10	13	7
Female	837	711	29	45	14	10	19	9
χ2		16.687	3.132	1.777	12.466	1.712	0.297	0.416
P		0.000	0.077	0.183	0.000	0.191	0.586	0.519

The causative agents were classified as dermatophytes (32.85%), yeasts (49.20%) and NDM (17.95%), and their distributions among different body sites are given in **Table 3** and **4**, including two dermatophyte species, two yeast species and two NDM. Positive cultures of dermatophytes were obtained from 430 patients: *Trichophyton* spp. (89.07%, 383/430, 95%Cl: 85.91-91.61%) were predominant, followed by *Microsporum* spp. (10.93%, 47/430, 95%Cl: 8.39-14.09%). In such instances, *Trichophyton rubrum* was the most common species, accounting for 60.93% of the dermatophytes in the different samples. Yeasts were isolated from 644 patients. The major pathogen was *Candida* spp. (93.01%, 599/644, 95%Cl: 90.86-94.73%), followed by *Trichosporon* spp. (6.99%, 45/644, 95%Cl: 5.27-9.14%). NDM was isolated from 235 patients, and *Aspergillus* spp. (90.21%, 212/235, 95%Cl: 85.87-93.44%) were the major pathogens, followed by *Fusarium* spp. (9.79%, 23/235, 95%Cl: 6.56-14.13%).

**Table 3:** Clinical types of superficial dermatomycosis and distribution of fungal pathogens.

Species	Tinea unguium(%)	Tinea pedis(%)	Tinea cruris(%)	Tinea corporis(%)	Tinea capitis(%)	Tinea manuum(%)	Tinea faciei(%)	Other SFIs(%)
Dermatophyte								
*Trichophyton rubrum*	213(62.47)	13(72.21)	11(61.11)	22(66.67)	2(12.50)		1(33.33)	
*T. mentagrophytes*	67(19.65)	3(16.67)	7(38.89)	4(12.12)	4(25.00)		1(33.33)	
*T. meginii*	1(0.29)							
*T. interdigitale*	26(7.62)	1(5.56)		1(3.03)				
*T. violaceum*					1(6.25)			
*T. gypreum*	4(1.17)					1(100.00)		
*Microsporum canis*	2(0.59)			3(9.09)	6(37.50)			
*M. gypseum*	28(8.21)	1(5.56)		3(9.09)	3(18.75)		1(33.33)	
NDM								
*Fusarium spp*								23(2.62)
*Aspergillus spp*								212(24.12)
Yeast								
*Candida spp*								599(68.14)
*Trichosporon spp.*								45(5.12)
Total(%)	341	18	18	33	16	1	3	879

*The table clarifying that the percentages represent sample composition, and the corresponding 95% CIs are provided in the main text.

**Table 4:** Clinical types of “other SFIs” and distribution of fungal pathogens.

	Toenails	Fingernails	Toenails and Fingernails	Feet	Glabrous skin	Groin	Scalp	Hands	Face	Total N(%)
*Candida spp*	144	203	156	15	31	16	4	19	11	599(68.15)
*Trichosporon spp*	22	7	2	11	1	1		1		45(5.12)
*Fusarium spp*	11	1	8	2	1					23(2.61)
*Aspergillus spp*	106	20	52	9	14	3		6	2	212(24.12)
Total	283	231	218	37	47	20	4	26	13	879(100)

“Other SFIs”: SFIs are caused by yeasts and non-dermatophyte filamentous fungi (NDFF).

*The table clarifying that the percentages represent sample composition, and the corresponding 95% CIs are provided in the main text.

**Table 3** describes the distribution of dermatophytes among different anatomic sites of infection. Tinea unguium, tinea corporis, tinea cruris and tinea pedis were the top four, with tinea unguium being the most prominent. *Trichophyton rubrum* (62.47%, 213/341, 95%Cl: 57.21-67.44%) was the predominant species in cases with tinea unguium, followed by *T. mentagrophytes* (19.65%, 67/341, 95%Cl: 15.78-24.19%) and *Microsporum. gypseum* (8.21%, 28/341, 95%Cl: 5.74-11.61%). **Table 4** shows the distributions of NDM and yeasts among different body sites. The main site infected with NDM and yeasts was the nails (83.28%, 732/879, 95%Cl: 80.67-85.60%). *Candida* spp. was the predominant agent in patients with onychomycosis (68.72%, 503/732, 95%Cl: 65.27-71.97%). The second most common species was *Aspergillus* spp. (24.32%, 178/732, 95%Cl: 21.35-27.55%). Taken all the body sites evaluated together, onychomycosis was the most prevalent SFI, and *T. rubrum*, *Candida* spp. and *Aspergillus* spp. were the most representative species.

## 4 Discussion

Superficial fungal infections are related to several predisposing factors, including geographic and climatic conditions; demography, such as age and gender; migration; socioeconomic conditions; lifestyle; and the environment (Segal and Elad, 2021). Infections of nails by dermatophytes (tinea unguium), yeasts or NDM are clinically almost indistinguishable; therefore, the general term onychomycosis is often used (Degreef, 2008). In this study, onychomycosis was the most commom form of SFI, irrespective of gender and causative pathogen. This is consistent with a retrospective study from Guangdong, southern China (Cai et al., 2016). Onychomycosis can infect both fingernails and toenails, but onychomycosis of the toenail is much more prevalent (Aggarwal et al., 2020; Khalifa et al., 2023). In our study, 1069 patients suffered from onychomycosis, with 431 patients infected in toenails, 265 patients infected in fingernails and 373 patients infected in both toenails and fingernails. Onychomycosis is usually associated with several predisposing factors, such as genetic predisposition, excessive foot sweating, trauma in the region, and the habit of not wearing sandals in public bathrooms (Silva-Rocha et al., 2017). Although it is not life-threatening, it can seriously affect a patient’s quality of life (Nenoff et al., 2014). Infected nails can cause local pain or abnormal sensations, which in turn can lead to difficulties with activities such as walking or fitting into shoes, and can impair social interactions (Lipner and Scher, 2019; Gupta et al., 2020).

The species distribution in cases of onychomycosis varies across studies. Approximately 90% of toenails and 75% of fingernail onychomycosis cases are caused by dermatophytes globally (Leung et al., 2020; Powell et al., 2022). *Trichophyton rubrum* is responsible for the majority of dermatophyte-associated infections (Kruithoff et al., 2023). This study revealed that among the SFIs caused by *Trichophyton rubrum*, tinea unguium had the highest prevalence, with a total of 213 cases, accounting for 81.30% of the total. The possible reason is that *Trichophyton rubrum* tends to invade nails, so when the proportion of superficial mycosis caused by *Trichophyton rubrum* is analyzed separately, the proportion of tinea unguium is highest. Moreover, we found that *Candida* spp. were the most prevalent species in cases of onychomycosis. This varies in different cities in China, e.g., *T. rubrum* was the most common species of onychomycosis in Guangdong and Nanchang (Cai et al., 2016). Previous studies have shown that dermatophytes are the major pathogens of onychomycosis in temperate countries, whereas *Candida* spp. represent a greater proportion of the population in the tropics and subtropics, which are characterized by a hot and humid climate (Chi et al., 2005). The warm and humid climate in Ningbo may provide favorable conditions for yeast growth. And Ningbo has a large population engaged in aquaculture and food processing, which involves prolonged hand immersion in water—a well-known risk factor for *Candida* onychomycosis (Le et al., 2020). We also observed that women were more likely to have onychomycosis than men were, probably because they performed wet housework and frequent manicures, which predisposed them to *Candida* onychomycosis. In addition, owing to the hidden location of the infection, male patients may not bring it up to their doctors as they see it as a trivial cosmetic issue, whereas women take their appearance more seriously. This is also why local patients have a high rate of visits after their fingernails are infected.

Beyond onychomycosis, our study revealed distinct patterns in other SFI types. Glabrous skin was the second most common infection site in our study. A comprehensive review revealed that *Trichophyton* spp. were the primary causative agents of glabrous skin superficial fungal infections worldwide in recent years (Chanyachailert et al., 2023). However, we found *Candida* spp. was the most prevalent species in our study. The similarity in pathogen proportions between nails and glabrous skin (**Table 3** and **4**) may reflect disease transmission patterns, as these infections are primarily acquired through person-to-person contact, particularly via direct exposure to infected individuals (Alharbi et al., 2022). Superficial fungal infection of the feet was the third most common form of SFI in our study. Numerous studies have shown that tinea pedis is most often caused by *Trichophyton rubrum*, and adult males have a greater prevalence of tinea pedis than females do (Nowicka and Nawrot, 2021; Leung et al., 2023; Nigam et al., 2024). However, our study found no significant gender difference in the prevalence of foot superficial fungal infections. This negative finding could be interpreted with caution due to the relatively small sample size and low proportion of male participants (36.06%). Potential contributing factors may include: (1) improved labor protection measures for local male workers that could reduce traditional risk exposures; and (2) increased fitness activities among women that may alter their exposure profiles.

The epidemiological pattern of dermatophytosis has been examined in several countries. In this study, we used routine procedures to identify pathogenic species of SFIs, which include evaluation of fungal colony structure, pigmentation and microscopic morphology. MALDI-TOF MS was used as a routine supplementary examination. There are limitations to those traditional methods. First, morphological characteristics vary frequently and are sometimes difficult to identify. Second, these methods can identify only the genera of pathogens. Third, fungal culture is time-consuming as it requires more than 2–3 weeks of growth for typical characteristics and the accuracy of the results depends on the expertise of the personnel (de Albuquerque Maranhão et al., 2019; Begum et al., 2020; Evrard et al., 2024). MALDI-TOF MS is based on the acquisition of a unique protein signature of each microorganism and its comparison to a well-established and validated MS spectral database. It has the advantages of excellent sensitivity, high throughput and simple operation. However, the use of MALDI-TOF MS for the identification of *Fusarium* spp. and dermatophytes is still poorly investigated as their good protein spectra are difficult to obtain. Moreover, this method still requires the cultivation of fungi, particularly dermatophytes (Calderaro and Chezzi, 2024; Lévesque et al., 2024). Thus, we used molecular methods such as sequencing of the rDNA-ITS region as a complementary means to identify atypical strains. In this study, we found that for patients with onychomycosis, doctors perform fungal fluorescence smears and fungal cultures on their nail debris, whereas for patients with tinea corporis, doctors usually do not perform fungal cultures on their infected skin debris. The reason for this might be that the course of treatment for onychomycosis is long, and the curative effect is strongly influenced by the type of fungus as well as laboratory investigations, whereas the treatment effect on tinea corporis relies more on the clinical presentation without a laboratory confirmation examination.

In conclusion, our study revealed that nails constitute the largest source of SFIs in Ningbo, China. Regarding onychomycosis, women are more likely to have onychomycosis than men are, and local patients have a high rate of visits after fingernail infection. Moreover, we found that *Candida* spp. were the most prevalent species in cases of onychomycosis, and *Trichophyton rubrum* was responsible for the majority of dermatophyte-associated infections. The high prevalence of *Candida* spp. in onychomycosis serves as a basis for future studies that incorporate susceptibility testing to guide empirical therapy in the context of rising resistance. These data may be useful for controlling superficial fungal infections in this specific population. However, there are few limitations in the study. First, in this study, the vast majority of specimens were from nail plates (81.67%). This dataset was unlikely to reflect the true epidemiology of all superficial mycoses. Future research should broaden the spectrum of sample types to encompass a wider range of clinical presentations, thereby generating more robust and comprehensive epidemiological data. Secondly, due to the retrospective nature of the study, data on concomitant diseases and potential risk factors were not systematically collected, which limits our ability to perform in-depth determinant analysis. Thirdly, his study did not include antifungal susceptibility testing or treatment outcome follow-up. Therefore, while we report the distribution of pathogenic fungi, we are unable to make any evidence-based inferences regarding the clinical efficacy of specific antifungal agents. Finally, this was a single center, retrospective study, and the subjects were mainly derived from the local population, so extending these results to other cohorts may require careful interpretation and further study.

## Data Availability

The original contributions presented in the study are included in the article/supplementary material. Further inquiries can be directed to the corresponding author.
